# Reprogrammed lipid metabolism in bladder cancer with cisplatin resistance

**DOI:** 10.18632/oncotarget.24229

**Published:** 2018-01-13

**Authors:** Min Young Lee, Austin Yeon, Muhammad Shahid, Eunho Cho, Vikram Sairam, Robert Figlin, Khae-Hwan Kim, Jayoung Kim

**Affiliations:** ^1^ Institute for Systems Biology, Seattle, WA, USA; ^2^ Department of Surgery and Biomedical Sciences, Cedars-Sinai Medical Center, Los Angeles, CA, USA; ^3^ University of California Los Angeles, Los Angeles, CA, USA; ^4^ Samuel Oschin Comprehensive Cancer Institute, Cedars-Sinai Medical Center, Los Angeles, CA, USA; ^5^ Department of Urology, Ga Cheon University College of Medicine, Incheon, South Korea

**Keywords:** lipidomics, cisplatin resistance, bladder cancer

## Abstract

Due to its tendency to recur and acquire chemoresistance quickly, bladder cancer (BC) remains to be an elusive and difficult disease. Patients with recurrent and chemoresistant BC have an extremely poor prognosis. One possible approach that may provide insightful and valuable information regarding resistance mechanisms is looking into the lipid metabolism of BC cells. Metabolism of lipids is essential for cancer cells and is associated with the regulation of a variety of key cellular processes and functions. This study conducted a comparative lipidomic profiling of two isogenic human T24 bladder cancer cell lines, one of which is clinically characterized as cisplatin-sensitive (T24S) and the other as cisplatin-resistant (T24R). Immunohistochemistry analysis revealed that expression of cytosolic acetyl-CoA synthetase 2 (ACSS2) is positively correlated with aggressive BC. Ultra performance liquid chromatography-mass spectrometry (UPLC-MS) analysis profiled a total of 1,864 lipids and levels of differentially expressed lipids suspected of being associated with cisplatin resistance were determined. In addition, we found that ACSS2 inhibition greatly perturbed levels of metabolites, including CE(18:1), CE(22:6), TG(49:1), and TG(53:2). This study broadens our current knowledge on the links between cisplatin resistance and lipid metabolism in aggressive BC and suggests potential biomarkers for identifying higher-risk patients.

## INTRODUCTION

Bladder cancer (BC) has undoubtedly impacted the lives of many, as it has become the fifth most prevalent type of cancer, with ∼76,000 new cases and ∼16,300 deaths per year worldwide [1–4]. Albeit treatment varies with each patient and tumor stage when found, the standard of treatment for BC usually involves surgical resection of the tumor followed by adjuvant chemotherapy with cisplatin [5]. While effective in some forms of cancer, cisplatin often loses its efficacy in BC patients. Resistance often builds up quickly, leaving fewer options for treatment. The mechanisms driving this increased resistance to cisplatin over time are generally unknown.

Since cancer initiation and progression is associated with changes in metabolism, investigation into the metabolomics of cancer could prove to be useful. In particular, dysregulated lipid metabolism, which regulates diverse classes of molecules that play critical roles in cellular energy, storage, and signaling [6], has been associated with aggressive forms of different cancers [7–10]. Furthermore, given that lipid metabolism is regulated by several oncogenic signaling pathways and is important for the initiation and progression of tumors [11], it is widely accepted that lipid alterations may serve as potential cancer biomarkers [12]. Lipidomic analysis aims to identify and quantify all the relevant lipids along with the goal of characterizing their interactions with other cellular components and functions, such as proteins or gene expression [13, 14]. Lipid and phospholipid metabolism also plays key roles in cellular motility, cell invasion, and tumor metastasis. Previous reports have shown that metabolic perturbation of phospholipids is associated with various cancer types [5, 15, 16], indicating that the composition of phospholipids may be critical for deciding the fate of tumor cells.

In this study, we hypothesized that a perturbed phospholipid metabolism is implicated in the more aggressive variants of BC. We sought to determine the lipid alterations underlying cisplatin resistance and address the lack of knowledge between metabolite alterations and drug resistance. Through a state-of-the-art mass spectrometry approach, the experimental results identified lipid metabolites specific to cisplatin-resistant BC cells.

## RESULTS

### Characterization of isogenic cisplatin sensitive- or -resistant bladder cancer cells

**Figure 1:**
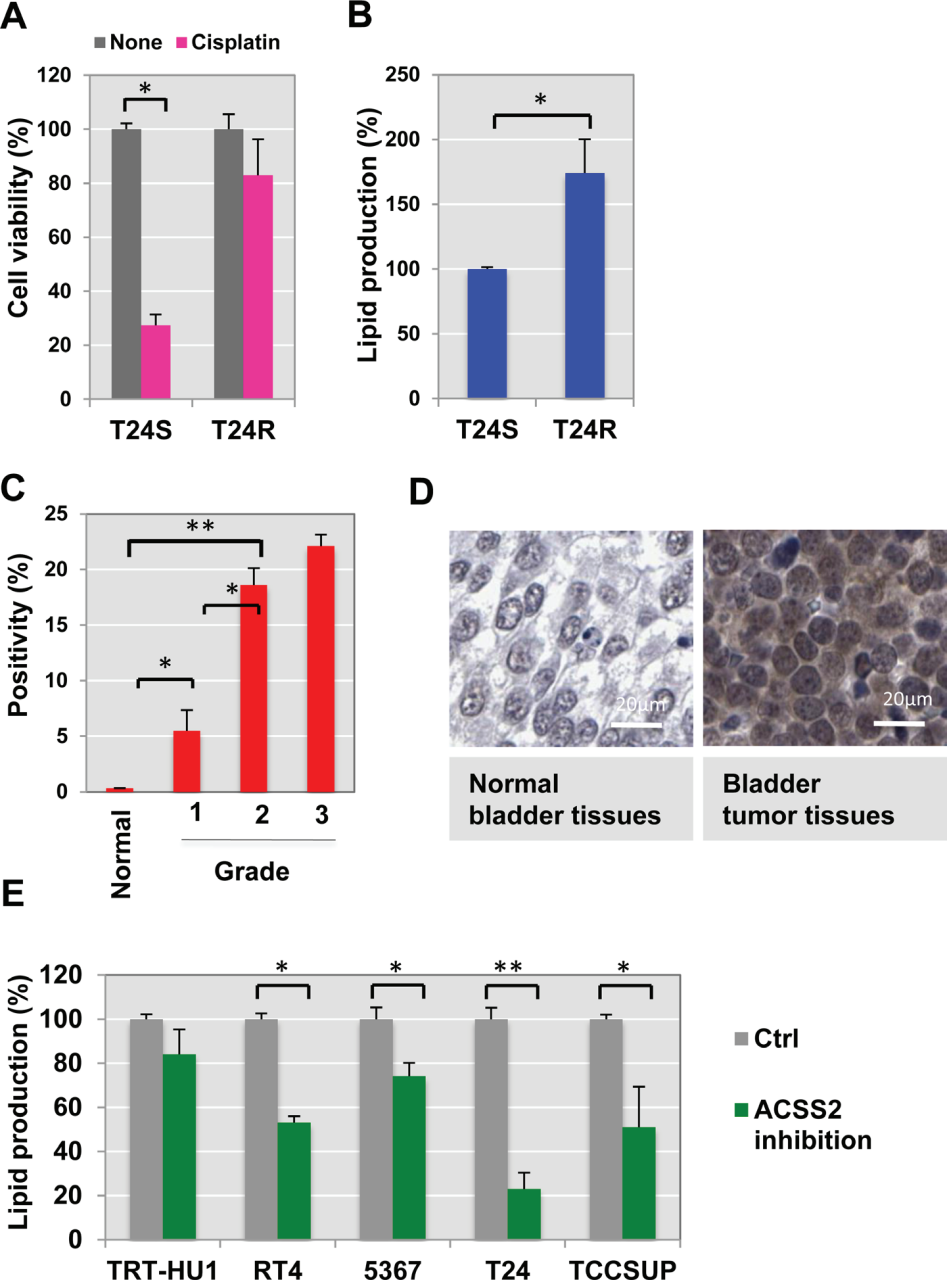
Cisplatin resistance is associated with ACSS2 (**A**) Cisplatin resistant T24R cells showed a delayed apoptosis in response to cisplatin treatment, compared to T24S. Cell viability was measured in the absence or presence of cisplatin media for 2 days. ^*^p < 0.05 (Student’s *t*-test). (**B**) Total lipid levels of T24R were compared to T24S cells. ^**^p < 0.005 (**C**–**D**) ACSS2 expression increased in bladder cancer tissues in comparison of normal bladder tissues. (**C**) The BC TMA was stained with anti-ACSS2 antibody (1:100) as described in the Materials and Methods section. The expression levels of ACSS2 were examined and quantified based on positivity of staining as previously described. (**D**) Images of one representative example of staining in normal noncancerous bladder tissue (left), and one representative example of staining in the paired bladder tumor tissues from same patients (right) were shown. (**E**) ACSS2 inhibition decreased total lipid contents in a series of BC cells. Cells were incubated with ACSS2 inhibitor-containing media for 20 hrs, which was followed by lipid measurement as described in Methods. ^*^p < 0.05, ^**^p < 0.005 (Student’s *t*-test).

To start identifying the cisplatin resistance-associated lipid metabolome, we utilized a paired cell culture system consisting of two isogenic cell lines, one of which was cisplatin sensitive (T24S) and the other resistant (T24R). Both cell lines were previously characterized in our laboratory [17]. Compared to T24S, T24R consistently exhibited a lower response to cisplatin-induced apoptosis (Figure 1A). The total lipid levels in T24R cells were approximately 170% of those in T24S cells, suggested that greater lipid production may be linked to cisplatin resistance (Figure 1B).

Prior studies have demonstrated that cancer cells have altered lipid metabolism [18, 19]. As a carbon source for producing fatty acids and cholesterol, acetate can be converted into cytosolic acetyl-CoA, which plays an important role in the TCA cycle [20–22]. The cytosolic acetyl-CoA synthetase 2 (ACSS2) mediates increased incorporation of acetate into lipids this processing. Given that a significant increase in the levels of ACSS2 has been found in a series of cancer types, such as melanoma, breast, ovarian, and lung cancers [21, 23–26], we speculated that ACSS2 expression may also be correlated with bladder cancer.

### ACSS2 expression is associated with bladder cancer aggressiveness

To determine the relationship of ACSS2 to lipid metabolism and cisplatin resistance at the molecular level, we performed immunohistochemical (IHC) staining of tumor microarrays using a commercially available ACSS2 antibody (Figure 1C–1D). This experiment revealed elevated ACSS2 expression in bladder tumor tissue cores. Our IHC analysis further confirmed that ACSS2 is more abundantly expressed in tumor samples of higher grades. These experimental results suggest that ACSS2 expression is significantly increased in BC cells and may also positively correlate with tumor grade.

Recently, high-throughput small molecule screenings of over 200,000 chemical compounds have identified inhibitors of ACSS2. One of the most potent and specific inhibitors is 1-(2,3-di(thiophen-2-yl)quinoxalin-6-yl)-3-(2-methoxyethyl)urea [25–27]. We were curious to see if inhibition of ACSS2 would have any effect on BC cells. We treated a set of normal bladder and BC cell lines (TRT-HU1, RT4, 5367, T24, and TCCSUP) with ACSS2 inhibitor for 24 h. Using this small ACSS2-inhibitory compound (MolPort), we found that total lipid levels of BC cell lines decreased, whereas those of normal cells (TRT-HU1) were not affected (Figure 1E).

### Identification of the lipids associated with cisplatin resistance

In order to determine the lipidomics profile of cisplatin-resistant BC cells, global and unbiased metabolomics analysis was performed. We identified differentially expressed lipids (DELs) by comparing BC cell lines T24R and T24S cells. Ultra performance liquid chromatography-mass spectrometry (UPLC-MS) detected a total of 1,864 lipids in both the positive and negative modes. The mass-to-charge (m/z) values, retention times, and abundance of the lipids are shown in Supplementary Table 1.

We measured 1,037 and 827 unique lipid species in the positive and negative modes, respectively. In the positive mode, 127 lipid species from cholesteryl ester (CE), ceramide (Cer), cholesterol, diacylglycerol (DG), glucosylceramide (GlcCer), lysophosphatidylcholines (LPC), phosphatidylcholine (PC), phosphatidylethanolamide (PE), sphingomyelin (SM) and triglyceride (TG) were identified. In the negative mode, 92 lipid species from Cer, fatty acyls (FA), GlcCer, LPC, PC, PE, phosphatidylinositols (PI), and SM were identified. Of interesting note, we found that essential structural components of cell membranes were major constituents of the identified lipids in both the positive (48 PC, 11 SM, and 44 TG) and negative (14 CER, 10 FA, 32 PC, and 21 PE) modes. DELs between T24S and T24R cells in the positive or negative ion mode are shown in Table 1 and Supplementary Tables 2 and 3.

**Table 1:** Differentially expressed lipids (DEL) between T24S and T24R in positive or negative ion mode

Mode	Identifier	Annotation	InChI Key	Species	m/z	RT	*P*-value	*Q*-value	Log_2_FC	Mean normalized intensity	
										S-	R-
Positive	10.30_719.57	CE (22:6)	VOEVEGPMRIYYKC -HNJOWPRISA-N	[M+Na]+	719.5729	10.30	0.0116	0.0911	1.4461	7881.9	21476.0
	4.93_768.55	PC (35:4)	OROZWUJCDDCYAU -IPUAOQJZSA-N	[M+H]+	768.5528	4.93	0.0204	0.0948	0.6450	18501.8	28933.0
	4.47_778.54	PC (36:6)	SPWBDEZMKCRQSX -NGPPOSSDSA-N	[M+H]+	778.5385	4.47	0.0048	0.0931	0.6402	14238.8	22192.0
	5.70_724.53	PE (p-36:4) / PE (o-36:5)	ADWDFBQPQIEGRZ -XBICFDGKSA-N	[M+H]+	724.5279	5.70	0.0002	0.0301	–0.6337	403765.9	260236.6
	6.39_752.56	PE (p-38:4) / PE (o-38:5)	ZTZQZGHJLWFLFQ -VZBWJDOASA-N	[M+H]+	752.5591	6.39	0.0142	0.0897	–0.8247	239093.0	134988.5
	6.40_778.57	PE (p-40:5) / PE (o-40:6)	HHQFKPJXVYWLLJ -ABYSKWQHSA-N	[M+H]+	778.5743	6.40	0.0185	0.0951	–0.6302	80510.8	52015.6
	7.15_813.68 _7.15_835.67	SM (d42:2)	DACOGJMBYLZYDH -GXJPFUDISA-N	[M+H]+ _[M+Na]+	813.6848 _835.6658	7.15	0.0070	0.0814	1.2411	204780.6	484070.9
	6.56_811.66 _6.55_833.65	SM (d42:3)	TXFLWJQVQCDUDZ -BRUGZULGSA-N	[M+H]+ _[M+Na]+	811.6624 _833.6505	6.56	0.0001	0.0558	4.3471	10492.5	213546.9
	11.16_829.73 _11.17_824.77	TG (48:0)	PVNIQBQSYATKKL -UHFFFAOYSA-N	[M+H]+ _[M+NH4]+	829.7256 _824.7705	11.17	0.0060	0.0894	–1.7587	190985.2	56439.4
	11.32_838.78	TG (49:0)	TTWJTJMWHOYBPQ -ANFMRNGASA-N	[M+NH4]+	838.7829	11.32	0.0262	0.0989	–1.6911	15051.5	4661.4
	10.96_841.73 _10.96_836.77	TG (49:1)	VYYGQDOPVVYUKW -UKFBYESTSA-N	[M+H]+ _[M+NH4]+	841.7263 _836.7709	10.96	0.0256	0.0993	–1.5552	64017.0	21784.1
	11.56_857.76 _11.57_852.80	TG (50:0)	MARPCPMDFOPPJX -UHFFFAOYSA-N	[M+Na]+ _[M+NH4]+	857.7562 _852.8018	11.57	0.0177	0.0989	–2.0145	151586.5	37517.4
	11.89_885.78 _11.91_880.84	TG (52:0)	SDNYRTVJOFMYIW -OIVUAWODSA-N	[M+H]+ _[M+NH4]+	885.784 _880.8354	11.90	0.0115	0.0920	–1.7912	96039.1	27748.3
	10.69_898.79 _10.67_903.74	TG (54:5)	OEJXMJPFOHYSIU -GRLFFVHSSA-N	[M+NH4]+ _[M+Na]+	898.7855 _903.7415	10.68	0.0191	0.0941	–1.4113	212399.1	79854.1
Negative	4.40_733.55	SM (d32:1)	KYICBZWZQPCUMO -PSALXKTOSA-N	[M+Hac-H]-	733.5478	4.40	0.0001	0.0027	1.1264	18915.2	41295.4
	7.15_871.69	SM (d42:2) B	DACOGJMBYLZYDH -GXJPFUDISA-N	[M+Hac-H]-	871.6894	7.15	0.0012	0.0212	1.2472	46562.3	110528.3

Annotated DELs with *q*-value less than 0.1 were shown. The full list of DELs was included in Supplementary Table 2 and 3. For each DEL, unique identifier given as RT, m/z, annotation identified by MS-Dial, InChI key, formula of the lipid, m/z, retention time (RT), *p*-value, *q*-value, log2-transformed fold-change (log_2_FC), and mean normalized intensities were shown.

### Inhibition of ACSS2 altered lipidomics profiles in cisplatin responsive bladder cancer cells

**Figure 2:**
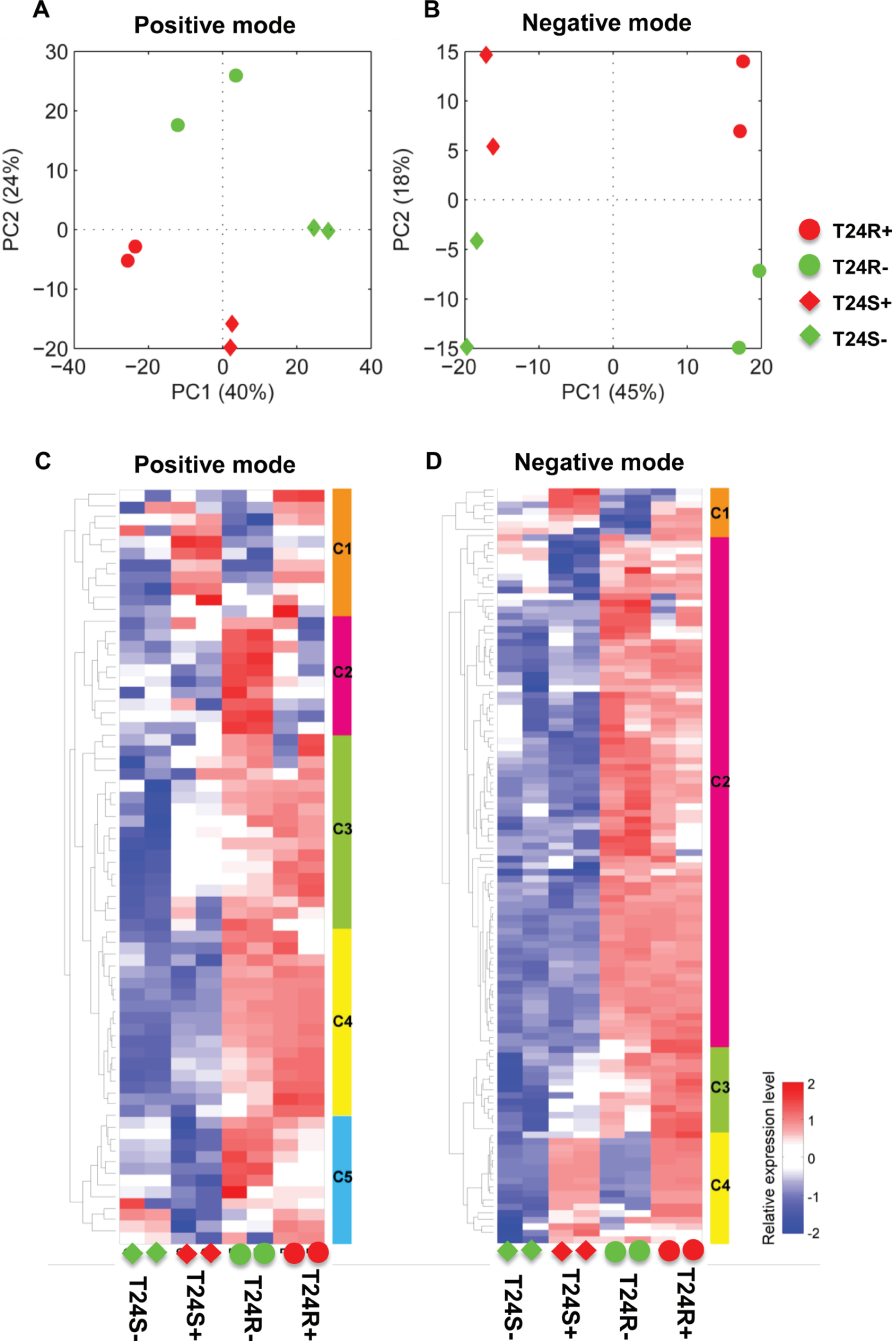
Differentially expressed lipids in T24R compared to T24S (**A**–**B**) Principal component analyses (PCA) of lipid profiles of T24S and T24R in the absence or presence of ACSS2 inhibitor were shown. The lipid profiles were acquired in (**A**) positive ion mode and (**B**) negative ion mode. S and R indicate cisplatin sensitive and resistant bladder cancer cell lines. The positive (+) and negative (-) symbols indicate treatment of ACSS2 inhibitor and vehicle, respectively. (**C**–**D**) Heatmaps showing differentially expressed lipids in T24R compared to T24S in the absence and the presence of ACSS2 inhibitor treatment. (**C**) DELs identified in positive mode, and (**D**) in negative mode.

We next attempted to understand the biological effect of ACSS2 inhibition. Given our data showing that T24 BC cells showed the biggest inhibitory effect on lipid levels by ACSS2 inhibition, we choose T24 cells for further experiments using ACSS2 inhibitor [25–27]. The overall lipidomic profiles showed larger differences between T24S and T24R cells after treatment with ACSS2 inhibitor than treatment with vehicles in both positive (Figure 2A) and negative (Figure 2B) modes. The full list of detected lipids in both the positive and negative modes are shown in Supplementary Table 1.

**Figure 3:**
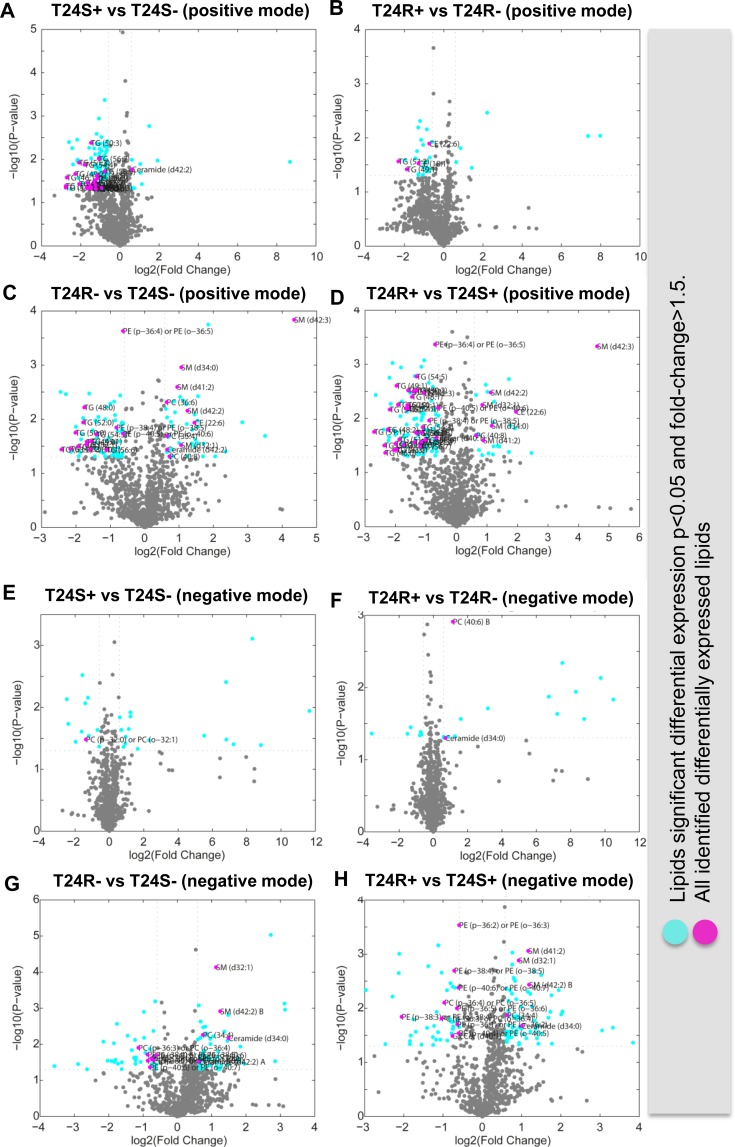
Volcano plots of cisplatin sensitive and resistant bladder cancer cell lines (**A**–**D**) Positive ion mode. (**A**) T24S+ and T24S–; (**B**) T24R+ and T24R–; (**C**) T24R– and T24S–; (**D**) T24R+ and T24S+. Cyan dots indicate the DEL (*p* < 0.05 and fold-change >1.5). Magenta dots indicate the identified DEL. (**E**–**H**) Negative mode. (**E**) T24S+ and T24S–; (**F**) T24R+ and T24R–; (**G**) T24R– and T24S–; (**H**) T24R+ and T24S+. Cyan dots indicate the DEL (*p* < 0.05 and fold-change >1.5). Magenta dots indicate the identified DEL. The x-axis and y-axis show log2-tranformed fold changes and log10-transformed *p*-values.

The difference between T24S– (T24S not treated with ACSS2 inhibitor) and T24S+ (T24S treated with ACSS2 inhibitor) was larger compared to the difference between T24R– (T24R not treated with ACSS2 inhibitor) and T24R+ (T24R treated with ACSS2 inhibitor) in the positive mode (Supplementary Table 2). The expression patterns of DELs showed distinct abundances of various lipid species between the cell lines and treatments in both the positive (Figure 3A) and negative modes (Figure 2B) (Supplementary Table 3). DEL between T24S+ and T24S– in the negative or positive mode are shown in Supplementary Tables 4 and 5, while those for T24R+ and T24R– in the negative or positive mode are shown in Supplementary Tables 6 and 7.

In our heatmap constructed from the data obtained from lipidomics analysis in the positive mode, Cluster 1 (C1) showed higher expression levels in ACSS2 inhibitor treated cell lines, regardless of cisplatin sensitivity (Figure 2C). Cluster 2(C2) and Cluster 5(C5) showed the highest expression in T24R– compared to others (e.g., CE (22:6)). Cluster 3(C3) and Cluster 4(C4) showed higher expression in T24R regardless of ACSS inhibitor treatment (e.g., Ceramide (d42:2), PC(36:6), PC(35:4), PC(40:8), SM (d42:3), SM(d42:2), SM(d41:2), SM(d32:1), and SM(d34:0)) (Figure 2C).

In contrast, the heatmap indicating lipidomics data in the negative mode is shown in Figure 2D. The most distinctive pattern was up-regulation in T24R, regardless of treatment with ACSS2 inhibitor. Cluster 2 (C2) included SM(d41:2), SM(d42:2), SM(d32:1), and PC(34:4). Cluster 1(C1) and Cluster 4(C4) showed up-regulation in ACSS inhibitor treated cells, regardless of cisplatin sensitivity (e.g., PC (40:6)). Cluster 3(C3) showed the lowest expression in T24S– and the highest expression in T24R+ (e.g., Ceramide(d42:2) and Ceramide(d34:0)) (Figure 2D).

### Distinct effects of ACSS2 inhibition on the lipid metabolome in cisplatin resistant bladder cancer cells

To further understand the metabolic consequences of ACSS2 inhibition, the lipid metabolite profiles of T24S+ and T24R+ were compared. Cisplatin-sensitive BC cell line (T24S) showed larger changes in lipid profiles compared to the resistant cell line (T24R). Among the identified lipids, we found that 23 TG lipid species were down-regulated in T24S+, compared to T24S–, in the positive mode (Figure 3A). Four lipid species, 2 CE and 2 TG, were downregulated in T24R+, compared to T24R–, in the positive mode (Figure 3B).

Compared to T24S in the positive mode, T24R had 42 lipid species that were upregulated and 68 species that were downregulated. Of these lipid species, 3 PC and 5 SM lipid species were upregulated and 3 PE and 12 TG lipid species were downregulated (Figure 3C). Compared to T24S+ in the positive mode, T24R + had 5 SM lipid species that were upregulated and 3 PE and 29 TG lipid species that were downregulated (Figure 3D).

Only a few identified lipid species were differentially expressed between T24S+ and T24S– (Figure 3E) and T24R+ and T24R– (Figure 3F). There were more differentially expressed lipids associated with cisplatin-resistance. Among the identified lipids, 6 PE lipid species were downregulated in T24R–, compared to T24S–, in the negative mode (Figure 3G). In addition, 2 PC and 7 PE lipid species were downregulated in T24R+, compared to T24S+, in the negative mode (Figure 3H).

**Figure 4:**
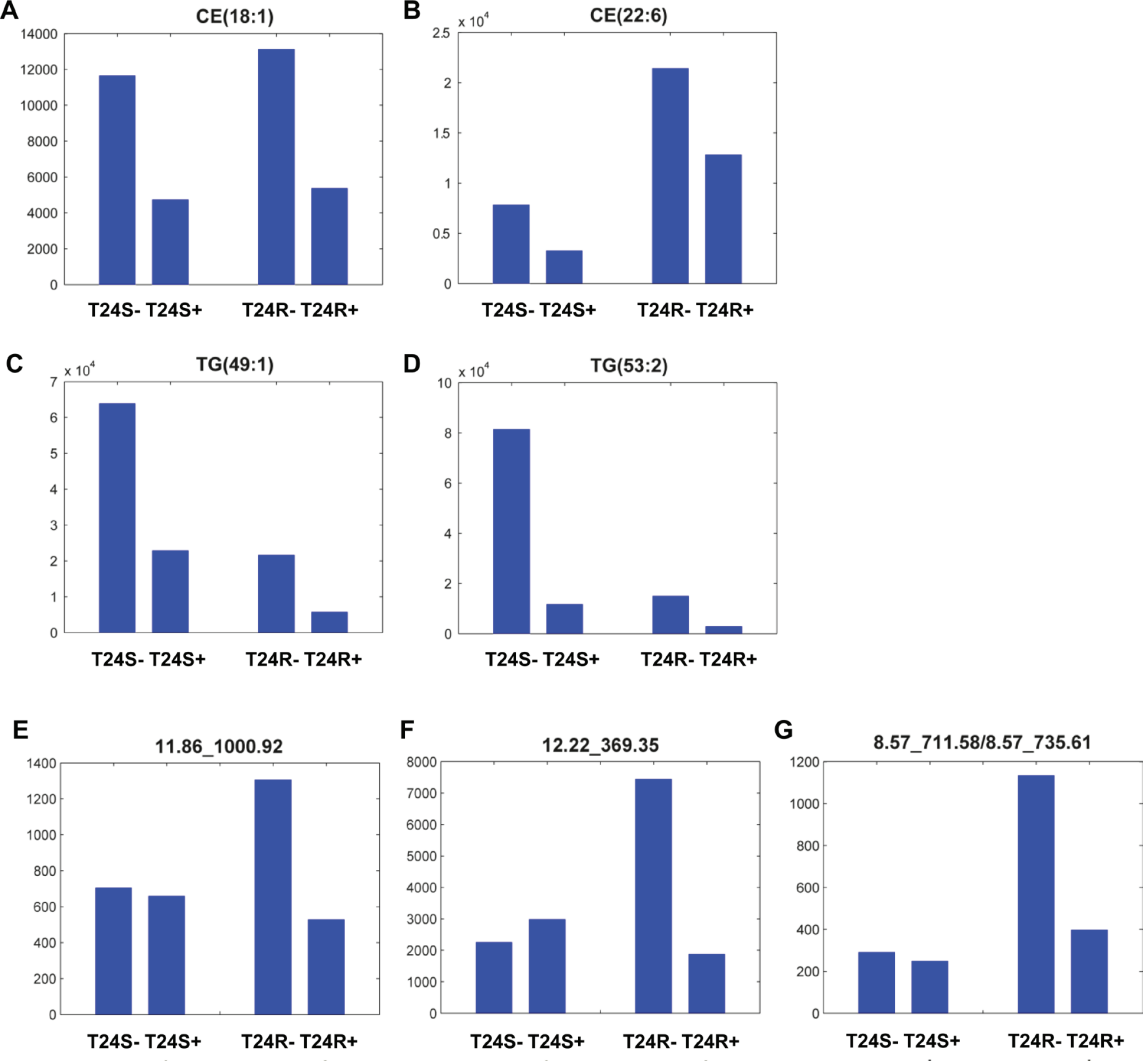
Lipid metabolites whose levels were decreased with ACSS2 inhibition (**A**–**D**) Among 27 lipid metabolites that significantly decreased by ACSS2 inhibitor in positive mode, four lipid species (**A**) CE(18:1), (**B**) CE(22:6), (**C**) TG(49:1), and (**D**) TG(53:2) were identified. (**E**–**G**) Examples of lipid metabolites specifically responsive to ACSS2 inhibitor treatment only in T24R cells. Three lipid species (**E**) 11.86_1000.92 in positive mode, (**F**) 12.22_369.35 in positive mode, and (**G**) 8.57_711.58/8.57_735.61 in negative mode showed statistically significant changes (*p* < 0.05 and fold-change >1.5) by ACSS2 inhibitor only in T24R cells. The lipid identifiers denote retention (RT) time and m/z.

These results show that the levels of most lipid metabolites specific to T24R were not significantly affected by ACSS2 inhibitor treatment (Figures 3A and 3B). Levels of metabolites, such as CE(18:1), CE(22:6), TG(49:1), and TG(53:2) were greatly perturbed by ACSS2 inhibition (Figures 4A–4D). In contrast, the expression levels of several unknown metabolites such as 11.86_1000.92, 12.22_369.35, and 8.57_711.58/8.57_735.61 decreased only in T24R cells, but not in T24S cells (Figures 4E–4G). Collectively, these findings suggest that cisplatin resistance is associated with defects in acetate and lipid metabolism.

## DISCUSSION

In this study, we sought to characterize the cisplatin resistant lipidome in BC. Through the integration of lipidomics and systems biology approaches, this study has broadened our understanding of the lipid biology associated with cisplatin resistance in BC. The experimental results also suggest potential lipid biomarkers and therapeutic targets.

Lipid metabolism is often disturbed in cancer cells and this is expected to result in differing lipid composition [28]. However, profiling of disease-related lipids specific to cancer is still underdeveloped, compared to those of genes and proteins. Lipidomics profiling aims to identify lipids using mass spectrometry and annotate them based on a metabolite database [12]. Other approaches that allow for analysis of lipids in cancer tissue include nuclear magnetic resonance (NMR), mass spectroscopy (MS) [12], matrix-assisted laser desorption and ionization (MALDI), and electrospray ionization (ESI) [29, 30], which allow for analysis of lipids in cancer tissue. Integration of lipidomic strategies in cancer research could generate new opportunities to gain insights into diagnosis, prognosis and prediction of therapies [14].

Experimental results from our study revealed a list of identified lipids that is heterogenous and belongs to several different classes. The original goal of this study was to determine the global lipid metabolome in cisplatin resistant BC. Although this is out of scope for this particular study, there has been much speculation regarding the potential biological significance of differential amounts of sphingomyelin (SM) and ceramide (CE) on membrane structure, permeability, and signaling platform formation. Several reports have suggested that strict distribution patterns of lipid species and enzymes determine cell fate through regulatory mechanisms. For instance, higher levels of SM are associated with drug resistance, possibly through alterations of membrane packing [31]. Sphingomyelinase, an enzyme that catalyzes the breakdown of SM into CE and phosphorylcholine, also induces apoptosis by altering the balance between SM and CE. Additionally, C16:0-ceramide has recently been reported as a principal mediator of obesity-related insulin resistance [32, 33]. Furthermore, in various cancer, phosphoglyercides, namely PC, PE, and PI, are upregulated [34, 35]. In particular, it is known that cholesterol and sphingolipids form specific planar microdomains, known as lipid rafts [36]. In cancer cells, these lipid rafts contain receptors for signaling proteins involved in oncogenic and apoptotic pathways [37]. Cholesterol is enhanced in the cell membrane of various cancer cells [38, 39]. Sphingolipids (sphingomyelin) are also involved in several cancer-related processes, such as proliferation, apoptosis and metastasis [40]. Therefore, by compiling lipidomic data, we could identify potential lipid signatures from a combination of different lipids, which could then be used to classify tumor samples from healthy controls.

Furthermore, our findings identified specific ACSS2-inhibiting lipid metabolites, such as CE(18:1), CE(22:6), TG(49:1), and TG(53:2) (Figure 4). Alterations in the levels of phospholipid metabolites, such as PC and PE, have often been considered as biochemical indicators of tumor progression or drug response [41–44]. Lipid perturbation in BC has been observed in a series of previous papers. From 40 paired bladder cancer and adjacent normal bladder tissues, Dill *et al.* found that levels of PI, PS, and FA were changed in BC tissues [45]. Using the DESI-IMS method, the authors found a significant increase of PS(18:0/18:1), PI(18:0/20:4) and FA(18:1) in bladder tumor tissues (*n* = 20), compared to healthy controls (*n* = 20). In the dog model BC (*n* = 4), levels of PS(18:0/18:1), PG(18:1/18:1), PI(16:0/18:1), PI(18:0/18:1), PS(18:1/18:1), PC(34:1), and PC(36:2) increased in BC tissue compared to normal [46]. Our study demonstrates that levels of CE(22:6), TG(49:1), and TG(53:1) *et al.* are significantly elevated in cisplatin-resistant BC cells compared to their cisplatin-sensitive counterparts. This conveys that these lipids may be actively involved in BC pathogenesis. Ceramide, a sphingomyelin byproduct and gangliosiode precursor, is potentially related to tumor growth and metastasis. Exogenous addition of a specific ganglioside mediated the epithelial-to-mesenchymal transition in BC cells [47], and also inhibited cell proliferation through inhibition of the EGFR signaling pathway [48, 49]. Furthermore, growth of bladder tumors was delayed by GM3 in the orthotopic bladder cancer model. N-glycolyl-GM3, which contains N-glycolylneuraminic acid instead of N-acetylneuraminic acid, was observed in colon and breast cancers and is suggested as a potential target for cancer therapy. We also found that TG species, which are composed of a glycerol molecule linked by ester bonds to three fatty acids with chain lengths of 49:1 or 53:2, were significantly decreased in cisplatin-resistant BC cells compared to cisplatin-sensitive control cells. TG (and diacylglycerols (DG)) plays an important role in cellular membranes and signaling pathways. TG also mediates the storage of fatty acids and energy, and provides the precursors needed for phospholipid biosynthesis.

Although these findings are quite exciting, this study has several limitations. While global lipidomics offer exciting technologies, we are aware that there are still constraints with data interpretation. Despite the fact that available databases for interpreting lipidomics profiles have progressed rapidly in recent years, many lipids and lipid metabolites are still functionally uncharacterized or completely unknown. In this current study, we attempted to extract and provide as much biological information as possible on the lipid metabolites identified. In addition, our study lacked further validation using independent and targeted analyses. A final major outstanding deficiency in this study was lipidomics profiling using clinical samples, which would have significantly strengthened our findings.

In summary, we used an untargeted lipidomics approach to depict a comprehensive picture of BC using both cisplatin-sensitive and resistant cell lines. We have confidently profiled a diverse lipid signature of 1,037 and 827 lipids for these cell lines. Specifically, differential lipid abundances were observed in molecular species that are known to have significant roles in regulating cancer progression. Based on this lipidomic study, adjusting lipid metabolism purposively may represent an optimal strategy in overcoming cisplatin resistance and improving cancer therapy. This study not only facilitates the development of new drugs against cisplatin resistance, but also provides a novel method in the clinical diagnosis of cisplatin-resistant bladder cancer.

## MATERIALS AND METHODS

### Cell culture and transfection

RT4, 5367, TCCSUP, and T24 BC cells were obtained and cultured, according to instructions provided by ATCC. Immortalized normal human bladder epithelial cells, TRT-HU1, were maintained as described previously [14]. Media was supplemented with 10% fetal bovine serum, 2% glutamine and 1% antibiotics (Invitrogen, Carlsbad, CA). Cells were maintained under a humidified atmosphere of 5% CO_2_ at 37° C. The TRT-HU1 cell line was constructed and extensively characterized in previously published papers. These cells lines tested negative for mycoplasma and all other infectious agents.

### Cell survival assay

T24S and T24R cell lines were incubated with 10 mM cisplatin for 2 days. Cell survival was determined by measuring cell viability using MTS reagents, according to the company’s protocols (Promega Corporation, Madison, WI).

### Lipidomics by charged surface hybrid column (CSH)-electrospray (ESI) quadrupole time of flight mass spectrometer (QTOF) MS/MS

Lipidomics and data analysis were conducted at the NIH West Coast Metabolomics Center (UC Davis).

#### Extraction

T24 cells were extracted as indicated in the following protocols [50], which was slightly modified from the earlier protocols [51]. The lipid extracts were separated using methanol and MTBE, which can separate lipids from proteins and other small polar hydrophilic molecules in a way such that the lipids were found in the top layer of a liquid-liquid separation, rather than in the bottom layer. Therefore, decanting the top layer ensured that the proteins or polar compounds did not contaminate the lipid extracts. We optimized the choice of internal standards and chromatographic conditions; e.g. by using toluene in the reconstitution solvent mixture to ensure that lipophilic components, like CE and TAGs, were transferred to the UHPLC column during the injection process.

#### Data acquisition

Data was acquired using the following chromatographic parameters: Column, Waters Acquity UPLC CSH C18 (100 mm length × 2.1 mm internal diameter; 1.7 μm particles); Mobile phase A, 60:40 acetonitrile:water + 10 mM ammonium formiate + 0.1% formic acid; Mobile phase B, 90:10 v/v isopropanol:acetonitrile + 10 mM ammonium formate + 0.1% formic acid; Column temperature: 65° C; Flow-rate: 0.6 mL/min; Injection volume: 3 μL; Injection temperature: 4° C; Gradient: 0 min 15% (B), 0–2 min 30% (B), 2–2.5 min 48% (B), 2.5–11 min 82% (B), 11–11.5 min 99% (B), 11.5–12 min 99% (B), 12–12.1 min 15% (B), 12.1–15 min 15% (B).

This chromatography method yielded excellent retention and separation of lipid classes (PC, lysoPC, PE, PS, TAG, ceramides) with narrow peak widths of 8–17 s. In addition, we saw very good within-series retention time reproducibility of better than 6 s absolute deviation. We used automatic valve switching after each injection, which was shown to reduce sample carryover for highly lipophilic compounds, such as TAGs from 29% to 0.1%. The valve switching occurred at 0.10, 11.60 and 13.00 minutes. At 0.1 minutes, the sample has flown through. Then at 11.60 minutes, the valve switching allows the mobile phase (99% B) to flow through the needle and flush out any highly lipophilic compounds. Finally, it switches at 13.00 minutes to re-equilibrate the needle to initial conditions. 100% isopropanol wash is used to wash the needle before every injection (after sample has been picked up).

The following mass spectrometry parameters were used: for positively charged lipids, such as PC, lysoPC, PE, and PS, an Agilent 6530 QTOF mass spectrometer was used with a resolution of *R* = 10,000; for negatively charged lipids, such as free fatty acids and phosphatidylinositols, an Agilent 6550 QTOF mass spectrometer set at *R* = 20,000 was used.

#### Identification and quantification of lipids

Raw data was processed through MS-DIAL [52]. First, peaks were detected and quantified for each sample. A peak table including RT, m/z, and intensity was constructed for each sample. Then, peak alignment across all samples was performed in four steps: (1) a reference peak table was constructed. The reference peak table started with one of the sample tables. All peaks from other sample tables were examined if they are in the reference table. If the peaks are outside the RT and m/z tolerance of 0.1 min and 0.025 D, they were inserted to the reference peak table. (2) All peak information including alignment ID, average RT, average m/z and intensities of all samples were associated to the reference peak table to generate a complete aligned peak table. (3) The aligned peaks with missing intensity information in all samples were filtered out. (4) In order to impute missing intensity values, MS-Dial searched a feature with a local maximum intensity with the same RT and m/z tolerance in step (1). When no feature was found to align in a sample, MS-Dial fills zero to indicate that the abundance of the peak was not measured.

### Normalization and identification of differentially expressed lipids

The normalization procedure was based on the sum of all peak heights for all identified lipids in each sample that were defined as mTIC. The normalization factor for a sample was calculated by dividing the average mTIC by the mTIC of the sample being assessed. The intensities of all the lipid species were then multiplied by the calculated normalization factor. Differentially expressed lipids (DEL) were identified based on a significance test of *P* < 0.05 from a two-sample *t*-test and if there was a fold change greater than 1.5. The *Q*-value, an FDR adjusted *p*-value, was computed for each *p*-value using the procedure introduced by Storey, 2002 [53]. Principal component analysis (PCA) was applied to summarize variations in the lipid profiles of different cell lines and the effect of treatment with ACSS1 inhibitor. The normalized intensities were transformed to a log scale with a base of 2. The missing values in a lipid species were estimated as the minimum value of the quantified values before PCA was applied. To cluster lipids that shared similar intensity profiles across conditions, hierarchical clustering was applied with Ward linkage and Euclidean distance as a similarity measure.

### IHC analysis using TMA

The tissue microarrays (TMA) were obtained from a commercial resource (Biomax, BL802). They contain 80 cases in 80 cores. Sixty cores were bladder cancer tissues, 10 cores were normal bladder tissues, and 10 cores were normal adjacent to tumors. The antibody specific to ACSS2 (D19C6) was purchased from Cell Signaling Technology (Beverly, MA, United States). For the antigen retrieval, we used a high pH method. For counterstaining, an Ultraview DAB Detection Kit (Ventana Medical Systems) was used.

### Slide annotation and digitalized IHC analysis

The slides were annotated and characterized using Leica Tissue IA 2.0 software (Copyright 2012 by SlidePath Ltd.). For quality control measures, hematoxylin and eosin (H&E) stained slides were used during the annotation. The ACSS2 slide was viewed with its corresponding H&E slide to ensure the annotation was done on the bladder tumor epithelium only. Stromal and structural tissues were cut off from annotation. A minimum number of cells measured per slide were set up as 100,000 cells/core.

Following annotations, analysis was done using the Leica Tissue IA 2.0 software. The Measure Stained Cells Algorithm was selected. Color definition preferences were defined, and algorithm input parameters were optimized by using several stained images of slides. The optimized algorithm was used for the analysis of all slides. Haematoxylin was set as the nuclear counter stain, and 3,3’Diaminobenzidine (DAB) was set as the nuclear, cytoplasmic, and membrane marker.

Parameters in the software are based on a grayscale. Zero is the minimum intensity (black), and 255 is the maximum intensity (white). We set the threshold range at 170 and 80 for positive and negative staining, respectively. The max nuclear window size radius was set to default and the nuclear area threshold was set to 0–500 mm^2^ (any nuclei or cells out of these range were cut off). The minimum percentage of stained area in order to be considered positive in the nucleus was set to 20%. The threshold for cytoplasmic staining was set at a higher range (160–22) because of the background contrast of the cytoplasmic counterstain and the antibody staining. In order for positive cytoplasmic staining to be detected, the threshold had to be set higher since the blue cytoplasmic staining was only slightly lighter than the antibody staining. The minimum percentage of stained area was set to 75%. After the annotation analysis, data of the nuclear h-score, % of positive nuclei, % of positive nuclear area, the cellular cytoplasmic h-score, and % of positive cytoplasmic staining were collected and visualized into box plots. Each annotated core had a minimum threshold of 100,000 cells to be analyzed. Three individual measurements were performed in a blinded manner without any information of clinical data. After computerized data analysis, positivity was collected from data for the % of positive nuclei in tissue, which was used for comparative graphing.

### Measurement of lipid levels in cells

Cells (1.2 × 10^6^/experimental group) were finely harvested in phosphate buffered saline (PBS) on ice, and total lipid levels were determined after lipid extraction using the Infinity Cholesterol Liquid Stable Reagent (Thermo Electron Corp., Waltham, MA).

### Statistical analysis

The mean of more than three replicates was used as the average. The *p*-values were calculated using a standard unpaired Student’s *t*-test for simple comparisons and *p* > 0.05 was considered statistically significant.

## SUPPLEMENTARY MATERIALS TABLES
















