# Supplementary material for: Reprogrammed lipid metabolism in bladder cancer with cisplatin resistance
Source: Oncotarget. 2018 Jan 13;9(17):13231–43. doi: 10.18632/oncotarget.24229 (PMC5862574; doi:10.18632/oncotarget.24229)
Supplement: Supplementary file 1 [file oncotarget-09-13231-s001.pdf]

Reprogrammed lipid metabolism in bladder cancer with cisplatin resistance

SUPPLEMENTARY MATERIALS

Supplementary Table 1: The list of detected lipids in positive or negative modes. See Supplementary_Table_1

Supplementary Table 2: Differentially expressed lipids between S- and R- in positive ion mode identified by *t*-test. For each DEL, unique identifier given as rt_mz, annotation identified by MS-Dial, InChI key, formula of the lipid, m/z, retention time (RT), *p*-value, *q*-value and log2-transformed fold-change (log_2FC) were shown. See Supplementary_Table_2

Supplementary Table 3: Differentially expressed lipids between S- and R- in negative ion mode identified by *t*-test. For each DEL, unique identifier given as rt_mz, annotation identified by MS-Dial, InChI key, formula of the lipid, m/z, retention time (RT), *p*-value, *q*-value and log2-transformed fold-change (log_2FC) were shown. See Supplementary_Table_3

Supplementary Table 4: Differentially expressed lipids between S+ and S- in positive ion mode identified by *t*-test. For each DEL, unique identifier given as rt_mz, annotation identified by MS-Dial, InChI key, formula of the lipid, m/z, retention time (RT), *p*-value, *q*-value and log2-transformed fold-change (log_2FC) were shown. See Supplementary_Table_4

Supplementary Table 5: Differentially expressed lipids (DELs) between S+ and S- in negative ion mode identified by *t*-test. For each DEL, unique identifier given as rt_mz, annotation identified by MS-Dial, InChI key, formula of the lipid, m/z, retention time (RT), *p*-value, *q*-value and log2-transformed fold-change (log_2FC) were shown. See Supplementary_Table_5

Supplementary Table 6: Differentially expressed lipids between R+ and R- in positive ion mode identified by *t*-test. For each DEL, unique identifier given as rt_mz, annotation identified by MS-Dial, InChI key, formula of the lipid, m/z, retention time (RT), *p*-value, *q*-value and log2-transformed fold-change (log_2FC) were shown. See Supplementary_Table_6

Supplementary Table 7: Differentially expressed lipids between R+ and R- in negative ion mode identified by *t*-test. For each DEL, unique identifier given as rt_mz, annotation identified by MS-Dial, InChI key, formula of the lipid, m/z, retention time (RT), *p*-value, *q*-value and log2-transformed fold-change (log_2FC) were shown. See Supplementary_Table_7
